# Distribution of axon diameters in cortical white matter: an electron-microscopic study on three human brains and a macaque

**DOI:** 10.1007/s00422-014-0626-2

**Published:** 2014-08-21

**Authors:** Daniel Liewald, Robert Miller, Nikos Logothetis, Hans-Joachim Wagner, Almut Schüz

**Affiliations:** 1 Max Planck Institute for Biological Cybernetics, Spemannstr. 38/41, 72076 Tübingen, Germany; 2 Department of Psychological Medicine, University of Otago, Wellington, New Zealand; 3 Anatomical Institute, University of Tübingen, Tübingen, Germany

**Keywords:** Axon calibre, White matter, Conduction time, Diffusion weighted imaging, Electron microscopy, Myelin

## Abstract

The aim of this study was to obtain information on the axonal diameters of cortico-cortical fibres in the human brain, connecting distant regions of the same hemisphere via the white matter. Samples for electron microscopy were taken from the region of the superior longitudinal fascicle and from the transitional white matter between temporal and frontal lobe where the uncinate and inferior occipitofrontal fascicle merge. We measured the inner diameter of cross sections of myelinated axons. For comparison with data from the literature on the human corpus callosum, we also took samples from that region. For comparison with well-fixed material, we also included samples from corresponding regions of a monkey brain (*Macaca mulatta*). Fibre diameters in human brains ranged from 0.16 to 9 $\upmu \hbox {m}$. Distributions of diameters were similar in the three systems of cortico-cortical fibres investigated, both in humans and the monkey, with most of the average values below 1 $\upmu$m diameter and a small population of much thicker fibres. Within individual human brains, the averages were larger in the superior longitudinal fascicle than in the transitional zone between temporal and frontal lobe. An asymmetry between left and right could be found in one of the human brains, as well as in the monkey brain. A correlation was also found between the thickness of the myelin sheath and the inner axon diameter for axons whose calibre was greater than about 0.6 $\upmu \hbox {m}$. The results are compared to white matter data in other mammals and are discussed with respect to conduction velocity, brain size, cognition, as well as diffusion weighted imaging studies.

## Introduction

The diameter of axons can differ by a factor of more than 100 within the mammalian nervous system. Axons in the cerebral cortex or the parallel fibres in the cerebellar cortex can be as thin as 0.1 $\upmu \hbox {m}$ (Sultan 2000), while axons of more than 10 $\upmu \hbox {m}$ diameter have been described in the saphenous nerve of the cat (Gasser and Grundfest 1939) and in the optic nerve of cats and horses (Guo et al. 2001), or axons of more than 15 $\upmu \hbox {m}$ diameter in the peroneal nerve of the cat (Hursh 1939), in the optic nerve of the bottlenose dolphin (Dawson et al. 1982), and the human spinal cord (Häggqvist 1936).

Fibre calibre is related to conduction velocity. In myelinated axons, conduction velocity increases approximately linearly with axon diameter (Gasser and Grundfest 1939; Hursh 1939). Thus, the range of axon diameters provides information about the range of conduction velocities and, together with the distances between origin and target of fibres, about conduction times (Caminiti et al. 2009; Tomasi et al. 2012; Innocenti et al. 2013).

Conduction times certainly play an important role not only for direct sensory and motor interactions with the environment, but also for cognitive functions carried out by the cerebral cortex. However, with the exception of the corpus callosum, not much is known about the range of fibre diameters in cortico-cortical connections, especially in humans. In this project, we therefore investigated the distribution of calibre of cortico-cortical axons in the white matter of human brains, based on electron-microscopic material. The rich connectivity of the cortex in itself is one of the main characteristics of this part of the brain (Braitenberg 1978) and provides the basis for higher cognitive functions, such as memory, learning, decision-making, and language. Connectivity up to a distance of a few millimetres takes place within the grey matter (Fisken et al. 1975; DeFelipe et al. 1985), most of it by way of slowly conducting axons, in view of the fact that most axonal profiles in the neuropil of the cortical grey matter are thin and unmyelinated (e.g. Peters et al. 1991; Braitenberg and Schüz 1991). We were interested to know the range of fibre calibre in the population of cortico-cortical fibres passing through the white matter and connecting the cortex to itself over large distances.

The human brain is exceptional by its size and by its particular cognitive abilities. Both properties raise questions about the organization of brain dynamics and conduction times. For example, it is noteworthy that every known oscillatory pattern of the extracellular field potential, such as the delta, theta, or gamma rhythm, is also found in every mammal investigated to date. Notably, not only the frequency band of the field potential, but also the duration and temporal evolution, as well as its behavioural correlations, are conserved across species. Some of this may be explained by local phenomena, such as interactions between excitatory and inhibitory neurons, or by membrane channel properties. However, some dynamical aspects, such as the propagation of slow oscillations over the cortex or synchronization of activity in distant regions, will involve distant cortical and/or subcortical connections and will depend on conduction times (for review see Buzsáki et al. 2013). For example, it could be shown in mice and cats that synchronization between hemispheres can be abolished by cutting the corpus callosum (Munk et al. 1995; Mohajerani et al. 2010). The dependency of synchronization on axonal delays between hemispheres could also be demonstrated in a simulation study by Ritz et al. (1994). Such dynamical constancies over species are remarkable in view of a volume difference of several thousand times which implies larger distances among the neuronal somata of interacting regions and an inevitable lengthening of their axons. Axonal length and volume increase much more rapidly than the number of neurons. Moreover, the increase in the number of neurons could rapidly increase the ‘synaptic path length’ (average number of monosynaptic connections in the shortest path between two neurons). To keep this length similar, two factors will play a role: an increase in the average number of synapses per neuron (Schüz and Demianenko 1995) and, in addition, the insertion of ‘short cut’ connections, maintaining the so-called small world-type network with brain size. While such a solution can effectively decrease path length within the neocortex, the increased lengths of the axons will lead to increased conduction times of action potentials. To compensate for these excessive delays, axon calibre and myelination might also be increased (also discussed in Innocenti et al. 2013). An indication that larger brains deploy more short cuts (long-range connections) and also, to a certain degree, larger calibre axons is that the volume of the white matter increases at 4/3 power of the volume of grey matter during the course of evolution (Zhang and Sejnowski 2000). While the white matter occupies only 6 % of the neocortical volume in hedgehogs, it exceeds 40 % in humans (Allman 1999). Another indication for time-preservation mechanisms is that the latency of sensory evoked responses in humans compared with rodents is only modestly increased compared to brain size difference (Buzsáki et al. 2013). It follows that size-invariant time parsing in neural networks depends on neuronal conduction velocity, and this in turns depends on axon calibre and myelination.

Numerically, increase in calibre may affect only a fraction of the axonal population. For the corpus callosum, comparative anatomical studies have shown that a large population of axons has similar calibres in different species and only few axons stand out by a substantial increase in diameter with brain size (e.g. Jerison 1991; Schüz and Preißl 1996). Moreover, studies of antidromic stimulations in cat, monkey and rabbit have shown that in all these species, conduction times in callosal and other cortico-cortical axons can differ considerably, even within neuronal populations connecting the same distant cortical regions (Miller 1975; Swadlow et al. 1978; Swadlow 2000). This can only be explained by a mixture of slow- and fast-conducting axons, i.e. of unmyelinated or thin myelinated axons and myelinated axons of large calibre. In addition, it is to be expected that—for technical reasons—electrophysiological recordings are often biased towards a sampling of fast-conducting axons (Miller 1994; Logothetis 2003), leading to an underestimation of the contribution of long conduction times in cortico-cortical fibres. Thus, anatomical quantification of axon calibres is an important contribution to assess the real distribution of conduction velocities.

The existence of thin axons in large brains is, on the one hand, imposed by constraints in volume increase (Ringo et al. 1994). However, it may well be that the resulting long conduction times are by no means just a necessary evil. On the contrary, the existence of a large range of conduction times in humans may have even opened up new cognitive abilities, as will be discussed in the last part of the discussion (see also Miller 1991; Caminiti et al. 2009; Tomasi et al. 2012). This idea has led to a theory on human cerebral laterality, based on an assumption of differences in the ranges of axonal conduction times between left and right hemisphere (Miller 1987, 1996). For this reason, we collected our anatomical data separately for each hemisphere.

An increasing interest in electron-microscopic data on axonal calibre also comes from investigators using diffusion weighted imaging (DWI) methods. Comparison with cytological data from white matter helps to improve and optimize these methods for tractography in humans (e.g. Makris et al. 1997; Wakana et al. 2003; Caminiti et al. 2013) and even for estimating fibre diameters in vivo. Differences in fractional anisotropy between left and right hemispheres have already been shown for the arcuate fascicle and for a region underneath the precentral gyrus, in the latter case related to handedness (Büchel et al. 2004). Fractional anisotropy depends on axonal calibre, among other variables.

Fibres connecting distant regions of one hemisphere with each other form five major fascicles which run through the depth of the white matter (e.g. Nieuwenhuys et al. 2008). We chose two regions of white matter where one can get access to three of these fascicles: (1) a dorsolateral region which is passed by the superior longitudinal fascicle, a long dorsal pathway connecting the various lobes with each other. It includes the arcuate fascicle which plays an important role in language (e.g. Rolheiser et al. 2011). (2) We also investigated white matter at its transition between frontal and temporal lobe. There the uncinate fascicle and inferior occipitofrontal fascicle form a compact bundle and connect the frontal lobe with the temporal and occipital lobe.

Since electron-microscopic material from post-mortem human brains is inevitably of low quality due to late fixation, for comparative purposes, we also included electron microscopic samples from regions in a freshly fixed monkey brain whose topography corresponded to the human samples.

This work has a direct link to Valentino Braitenberg. His own work was strongly based on quantitative neuroanatomy as a tool to understand the mechanisms behind brain functions, an approach which proved to be very successful in his hands. The present study continues this ambition. In fact, the initial spark for this study resulted from a cooperation with him in which we estimated the number of fibres in the various intrahemispheric cortico-cortical fascicles of the human white matter (Schüz and Braitenberg 2002). For this estimate, the density of fibres in these fascicles was needed, but to our knowledge, no such data existed. So we used the data by Aboitiz et al. (1992) on the corpus callosum, assuming that these can be generalized to other cortico-cortical, long-range systems. To what degree this is the case will also be answered in this study. For this purpose, we included here also samples from the corpus callosum. We dedicate this paper to the memory of Valentino.

## Methods

### Human brains

**Fig. 1:**
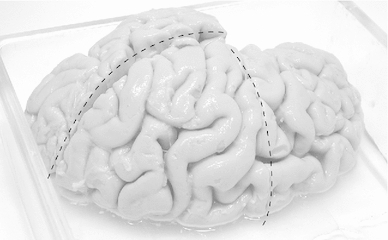
Left hemisphere of brain 3, indicating the course of the two macroscopic sections by *stippled lines*

**Fig. 2:**
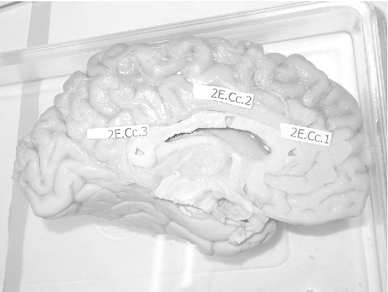
Brain 3, location of the three samples taken from the corpus callosum of brains 2 and 3

**Fig. 3:**
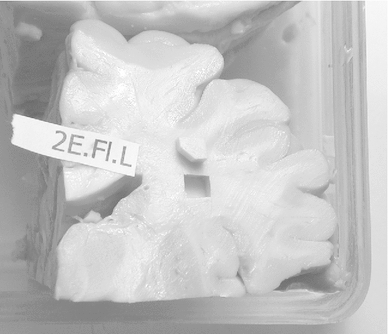
Coronal section through the left hemisphere of brain 2 (see Fig. 1), rostral side of the posterior part of the hemisphere. It shows the location of the sample cut out for further investigation. One edge of the sample has been chamfered to facilitate orientation of semi-thin sections

Human brains were taken from donors who had donated their bodies for education of medical students (dissection course). Their consent included use of tissue for scientific purposes. The donors had no neurological diseases, but other than age, cause of death and sex, no detailed medical history was known because the treatment of donors included a strict anonymization process. We had at our disposal material from three adult human brains. Brains No 2 and 3 were male brains from persons who died at the age of 89 and 74 years, respectively. We had no information on brain No 1 except that it was from a similar age group. Fixation by infusion with 4 % formaldehyde through the femoral artery had been carried out $\le$48 h post-mortem. The interval from fixation to dissection was between 6 and 18 months. Post-fixation time after removal of the brains was about 6 months in case of brain 1, 5 weeks in case of brain 2 and 1 week in case of brain 3. The hemispheres were then separated by a cut through the midsagittal plane and kept in fixative for another 48 h. Then, each hemisphere was cut into two pieces by way of an oblique horizontal section along the lateral fissure (Fig. 1) in order to get access to a cross section through the uncinate and inferior occipitofrontal fascicle. They merge into one tract at their transition between frontal and temporal lobe. A block of tissue was cut out at this transition zone. Also, three blocks were cut out of the corpus callosum (genu, truncus and splenium; Fig. 2). We then made a frontal section through the posterior part of the frontal lobe, aiming at cross sections through the superior longitudinal fascicle (Fig. 1). The superior longitudinal fascicle runs lateral to the corona radiata which radiates from the internal capsule. We therefore took a block of tissue lateral to the extrapolated dorsal extension of the internal capsule in the white matter (Fig. 3). The tissue blocks were then put into fixatives I and II (see below). In the case of the uncinate/inferior occipitofrontal fascicle and the superior longitudinal fascicle, the blocks had a relatively large areal size of up to 0.5 and $0.8\hbox { cm}^{2}$ (e.g. Fig. 3). These were cut further into 2 or 3 pieces before osmication.

Further treatment of tissue (washing in glucose-containing buffer, post-fixation in 2 % $\hbox {OsO}_{4}$ for 2–3 h) was done according to Palay and Chan-Palay (1974), with the following modifications: after osmication, the tissue was washed for $3 \times 2$ min in cold 0.1 M sodium acetate buffer, pH 7.4; then, the tissue was transferred into 0.5 % uranyl acetate in distilled water for 30 min in the refrigerator. Dehydration was carried out with ethanol and acetone, and the tissue was embedded in Epon/Araldite.

### *Macaca mulatta*

The monkey brain which we had at our disposal was from a 5-year-old male *Macaca mulatta* which had been perfused at the end of a terminal electrophysiological experiment within a project approved by the local authorities (Regierungspräsidium Tübingen) and in full compliance with the Directive of the European Community (86/609/EEC) for the protection of animals used for experimental and other scientific purposes. The animal was perfused for 20 min with saline in 0.1 M phosphate buffer (Sørensen buffer, pH 7.4) at $37^{\circ }\hbox {C}$, containing Heparin (100 mg/L), then with fixative solutions according to LaMantia and Rakic (1990). Fixative I contained 1.25 % paraformaldehyde, 1.25 % glutaraldehyde and 4 mL of 0.5 % $\hbox {CaCl}_{2}$/Litre, and Fixative II contained 2.5 % paraformaldehyde, 2.5 % glutaraldehyde and 4 mL of 0.5 % $\hbox {CaCl}_{2}$/Litre. For both fixatives, we used 0.1 M Sørensen buffer at pH 7.4. The first fixative was applied for 10 min, and the second for 25 min. The brain was left for 7 days in situ in the refrigerator in the first fixative. Then, the brain was taken out and left for another 4 days in the first fixative in the refrigerator. Pieces of tissue were cut out from locations corresponding topographically to those of the superior longitudinal and the uncinate/inferior occipitofrontal fascicle in the human brains, as well as from the truncus of the corpus callosum. The pieces were then washed in buffer, osmium-stained and embedded in Epon/Araldite as described above for the human brains.

From the various blocks, we made semi-thin sections (1 $\upmu \hbox {m}$ thick) and stained them with Azur-II-Methylene blue. Sectioning was orthogonal to the course of the fibre tracts, i.e. in the corpus callosum in the sagittal plane, in the uncinate/inferior occipitofrontal bundle in an oblique horizontal plane and in case of the region of the superior longitudinal fascicle in the frontal plane. In all cases, in semi-thin sections, we selected those regions in which all or most fibres were indeed running orthogonal to the section. This was particularly important in the region of the superior longitudinal fascicle the position of which can only be guessed topographically and which may, in addition, be crossed by fibres running in other directions. In cases where we had several blocks from one region, we chose the one which showed the most orthogonally running fibres.

### Sampling

The block from each region was then trimmed accordingly, and thin sections were cut for electron microscopy. They were mounted on mesh grids and contrasted with uranyl acetate and lead citrate. The sections now had an areal size of about $1.2\, \hbox {mm}^{2}$. On one section from each block, 8 samples were taken, i.e. 8 pictures at a primary magnification of 880 (Zeiss EM 912), covering an area of 13 $\upmu \hbox {m} \times 13\,\upmu \hbox {m}$ each. To avoid any bias in sampling towards smaller or larger diameters, the locations of the 8 samples were chosen at low magnification, each in the centre of a mesh. A sampling area was discarded and replaced by another only if, at high magnification, it turned out that we had hit a glia cell body or a blood vessel which would fill a large part of the sampling area. Samples were distributed over a region of about $1\,\hbox {mm}^{2}$. The total area investigated from each region was 1,352 $\upmu \hbox {m}^{2}$. Depending on the average calibre of the axons, such an area contained between 91 and 1,681 myelinated axonal profiles (see Table 1), whose diameters were measured.

**Table 1:** Mean value, standard deviation (SD), median, maximum and minimum value of axonal diameters (inner diameter of myelinated axons) in $\upmu \hbox {m}$; $n$ number of axons measured

	Mean/SD	Median	max	min	$n$
Human brain 1					
Sup. longitudinal fascicle					
Left	0.73 $\pm$ 0.55	0.57	3.73	0.19	210
Right	1.34 $\pm$ 0.9	1.14	4.0	0.24	91
Uncinate/inferior occipitofr. fasc.					
Left	0.61 $\pm$ 0.42	0.47	3.57	0.22	303
Right	0.54 $\pm$ 0.28	0.46	1.74	0.17	409
Human brain 2					
Sup. longitudinal fascicle					
Left	0.82 $\pm$ 0.64	0.64	4.58	0.19	165
Right	0.72 $\pm$ 0.49	0.59	4.79	0.21	365
Uncinate/inferior. occipitofr. fasc.					
Left	0.56 $\pm$ 0.32	0.48	2.01	0.19	402
Right	0.6 $\pm$ 0.38	0.51	4.42	0.17	414
Corpus callosum					
CC1	0.67 $\pm$ 0.42	0.53	2.56	0.17	389
CC2	0.67 $\pm$ 0.44	0.53	3.16	0.19	431
CC3	0.74 $\pm$ 0.47	0.59	2.8	0.24	250
Human brain 3					
Sup. longitudinal fascicle					
Left	0.63 $\pm$ 0.39	0.51	2.7	0.19	268
Right	0.67 $\pm$ 0.39	0.55	2.97	0.19	380
Uncinate/inferior occipitofr. fasc.					
Left	0.45 $\pm$ 0.28	0.36	2.54	0.16	640
Right	0.47 $\pm$ 0.28	0.38	2.09	0.17	762
Corpus callosum					
CC1	0.73 $\pm$ 0.45	0.58	2.43	0.19	376
CC2	0.64 $\pm$ 0.42	0.51	3.03	0.19	465
CC3	0.7 $\pm$ 0.56	0.51	5.13	0.18	451
*M. mulatta*					
Sup. longit. fasc.					
Right	0.51 $\pm$ 0.18	0.48	2.67	0.19	1681
Uncinate/inferior occipitofr. fasc.					
Left	0.78 $\pm$ .46	0.69	4.13	0.18	445
Right	0.55 $\pm$ 0.33	0.48	4.25	0.16	595
Corpus callosum					
CC2	0.69 $\pm$ 0.45	0.58	2.1	0.21	732

**Fig. 4:**
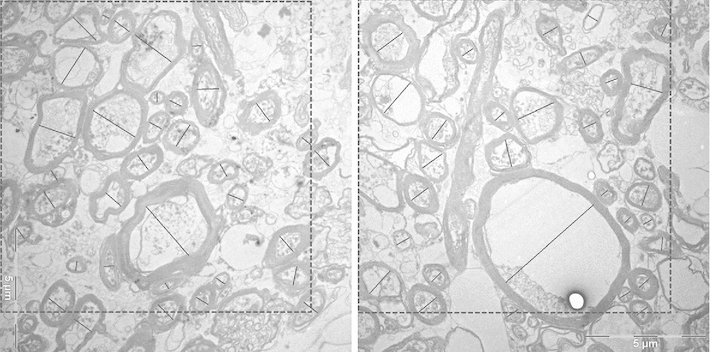
Electron micrographs from the superior longitudinal fascicle of human brain 2, illustrating the selection of profiles. The *broken lines* indicate the borders of the evaluated area in each electron micrograph. Profiles extending over the *upper* and *left borders* are discarded. The *black lines* within the profiles show how their diameters have been measured. *Bars* 5 $\upmu \hbox {m}$

Unmyelinated axons could not be identified with certainty in our human material, due to disintegration after late fixation. Thus, all our measurements (and also those in the monkey) were made on myelinated axons. Although the myelin sheath can also be affected by the late fixation, as can be seen in our micrographs, the myelin is usually easily recognizable and represents the original diameter of the axon, even if the axon itself may have started to disintegrate. The diameter of axons was measured within the inner borders of the myelin sheath, thus excluding the myelin sheath itself. Since profiles are often not circular but elliptic or elongated, depending on the angle with the plane of section, the longest diameter perpendicularly to the long axis of the profile was measured as in Partadiredja et al. (2003). Profiles of myelinated axons which intersected the right or the lower border of the sampling area were included, and those extending over the other two borders where discarded, as were profiles which had an awkward shape in which a diameter could not be defined, as well as profiles which ran more or less in parallel to the section. Profiles which could not be identified with high probability as axons were also discarded. In the few remaining questionable cases, the following additional criteria were applied: profiles with only a very thin dark membrane and no internal structure were excluded, as were profiles in which the membrane could not be clearly delimited from internal structures. Examples of measurements and selection of profiles are given in Fig. 4.

To test the statistical significance of differences between hemispheres or between human and monkey data, we used the test of Kolmogoroff and Smirnoff, based on cumulative frequencies of values within the 0.1 $\upmu \hbox {m}$-classes, which were used for the histograms in Figs. 8, 9, 10 and 11.

## Results

**Fig. 5:**
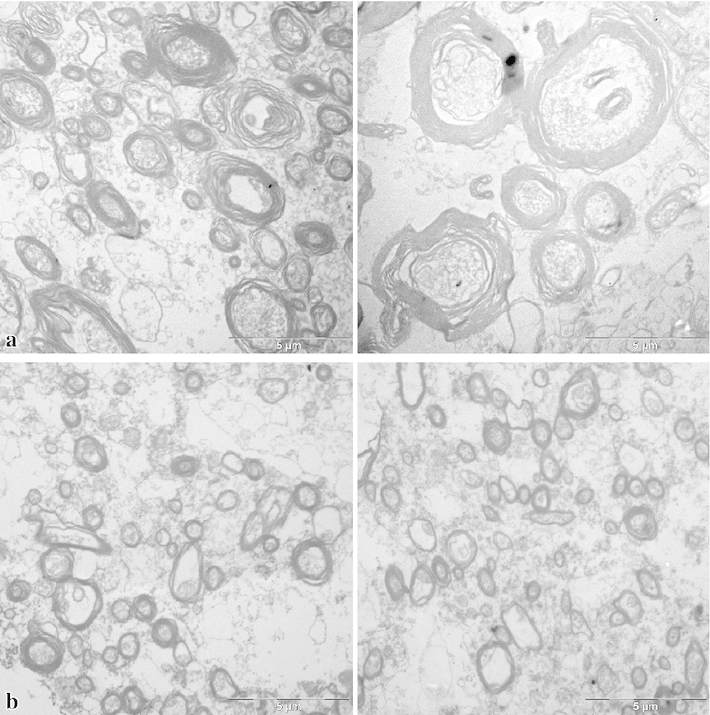
Electron micrographs from human brain 1, **a** from the superior longitudinal fascicle, **b** from the uncinate/inferior occipitofrontal fascicle. *Left* left hemisphere, *right* right hemisphere. *Bars* 5 $\upmu \hbox {m}$

**Fig. 6:**
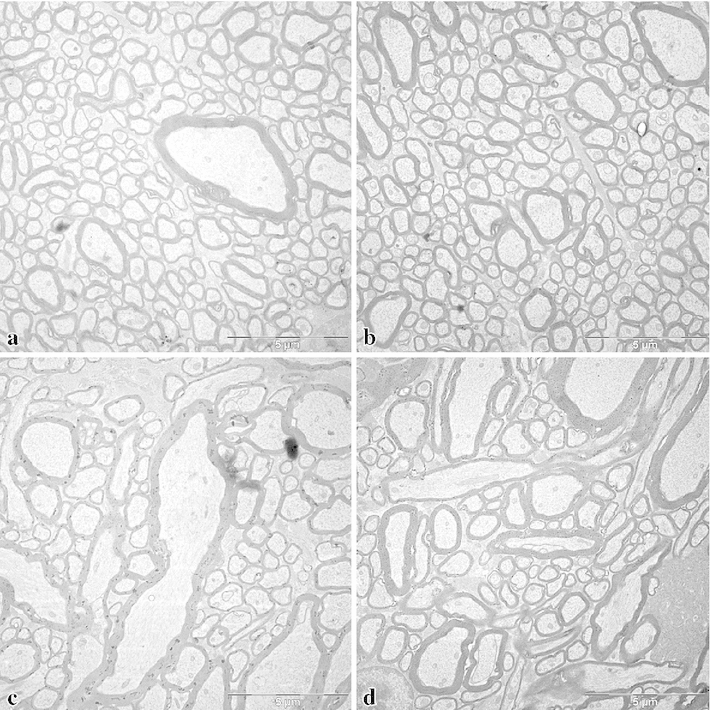
Electron micrographs from the region of the superior longitudinal fascicle of the macaque brain (*M. mulatta*). **a** and **b** show regions where all fibres run orthogonally to the frontal sections, **c** and **d** show a neighbouring region with fibres running also in other directions. This region was not included in the measurements, but is shown here for illustrating different aspects of the cortical white matter. *Bars* 5 $\upmu \hbox {m}$

**Fig. 7:**
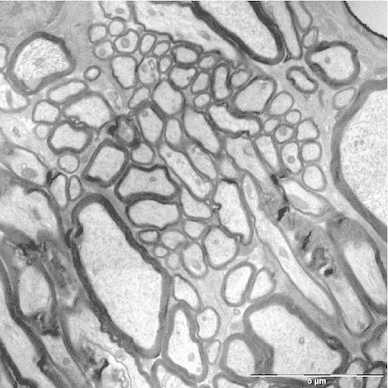
Electron micrograph from the corpus callosum of the macaque, showing the large range of diameters. *Bar* 5 $\upmu \hbox {m}$

Examples of electron micrographs from the white matter are shown in Figs. 4 and 5a (region of the superior longitudinal fascicle) and Fig. 5b (region of the uncinate/inferior occipitofrontal fascicle) from the human brains and in Figs. 6 and 7 from the monkey brain. As expected, the human tissue is less well preserved than that of the monkey, as evident from the debris between the axons and partly also within the lumen of the axons, as well as the detachment of membranes from each other in some of the myelin sheaths. In spite of the lower quality of the human tissue, myelinated axonal profiles can be well identified in most cases and the shape of the myelin sheath is usually preserved well enough to indentify the inner diameter. The pictures illustrate the wide range of diameters in both monkeys and humans.

**Fig. 8:**
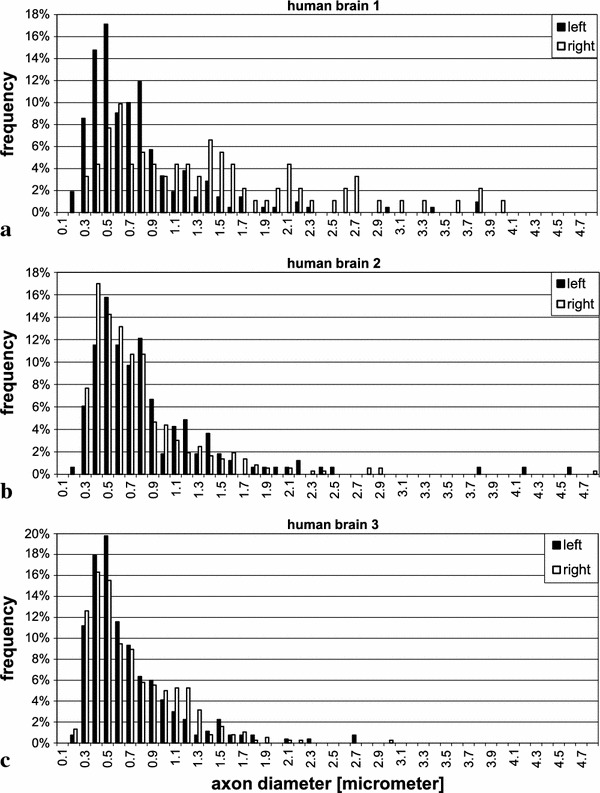
Frequency distribution of the diameters of myelinated axons in the superior longitudinal fascicle of the three human brains in the *left* and *right hemisphere*

**Fig. 9:**
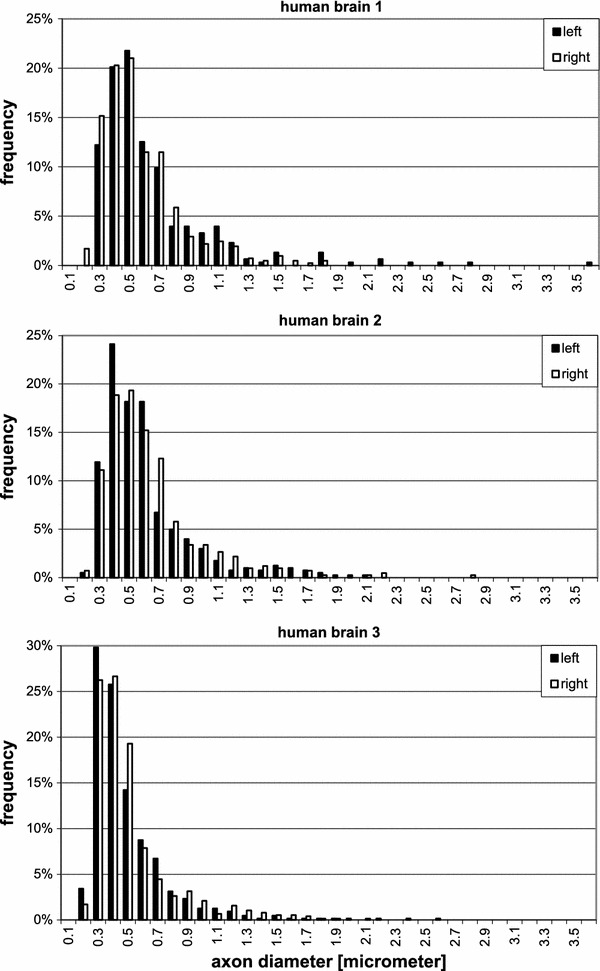
Frequency distribution in the uncinate/inferior occipitofrontal fascicle in the three human brains

**Fig. 10:**
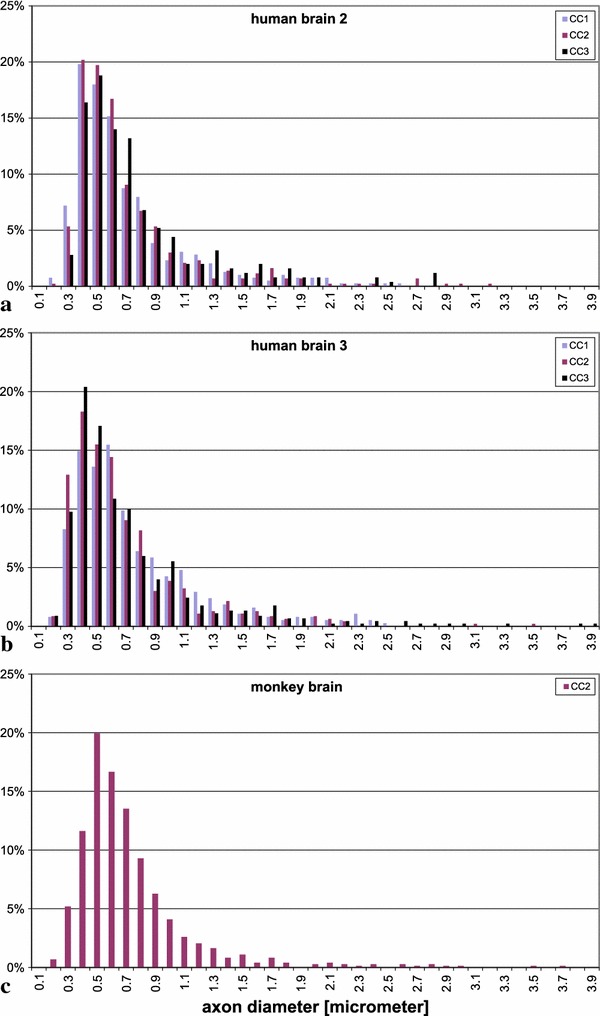
**a** and **b** Frequency distribution of the diameters of myelinated axons in three different locations of the corpus callosum in human brains 2 and 3 (CC1 genu, CC2 truncus, CC3 splenium). The largest value in human brain 3 is 5.13 $\upmu \hbox {m}$ and is not contained anymore in the diagram. **c** Axon diameters from the truncus of the corpus callosum of the rhesus monkey

**Fig. 11:**
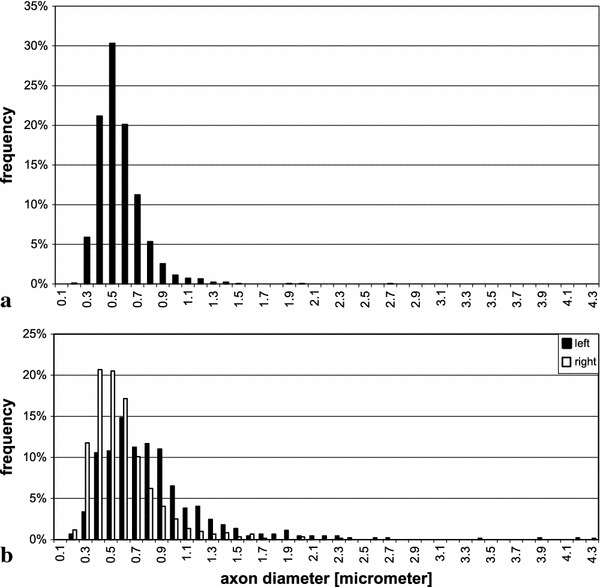
Frequency distribution of the diameters of myelinated axons in the rhesus monkey. **a** region of the superior longitudinal fascicle, *right hemisphere* only, **b** region of the uncinate/inferior occipitofrontal fascicle, both hemispheres

Table 1 shows the results of the measurements in the various regions of the individual brains and separately for both hemispheres. All data refer to inner diameter of myelinated axons, i.e. not including the myelin sheath. This also applies to all diagrams in Figs. 8, 9, 10 and 11. In both monkey and humans, most average and median values are smaller than 1 $\upmu \hbox {m}$, that is, slow-conducting axons predominate, and in all cases, distributions have a positive skew due to a small number of thick and very thick axons. The largest calibre axon we found in the human material within these cortico-cortical bundles had an inner diameter of 9 $\upmu \hbox {m}$ (Fig. 13). Results in more detail are as follows:

### Human brains

- *Region of the superior longitudinal fascicle (SLF)*. This fascicle is composed of four branches: SLF I, II and III, as well as the arcuate fascicle. Comparison of the location of our samples (Fig. 3) with the DT-MRI study in human brains by Makris et al. (2005) indicates that our samples are located in branch II and may also contain fibres of the arcuate fascicle the horizontal part of which runs together with this branch.

The diagrams in Fig. 8 show the distribution of axon diameters in this region for the three human brains. The diameters in our samples ranged from 0.19 to $4.79\,\upmu \hbox {m}$, with averages between 0.63 and $1.34\,\upmu \hbox {m}$ for the six hemispheres. In human brains 2 and 3, no significant difference between hemispheres could be detected. In human brain 1, a significant difference between the samples from left and right hemisphere could be seen, the right side showing significantly more fibres of larger calibre ($p<0.001$). The two pictures in Fig. 5 a are representative examples from the two hemispheres of this brain.

(b) *Region of the uncinate/superior occipitofrontal fascicle*. The diagrams in Fig. 9 show the distribution of diameters in this region for the three human brains. The values range between 0.17 and $4.42\,\upmu \hbox {m}$, with averages in the 6 hemispheres between 0.45 and $0.6\,\upmu \hbox {m}$, which were thus smaller than in the superior longitudinal fascicle ($p<0.01$). No significant difference between left and right could be found in the human brains.
(c) *Corpus callosum*. Figure 10a, b shows the distribution of axon diameters in the corpus callosum of brains 2 and 3. The values ranged between 0.17 and $5.13\,\upmu \hbox {m}$, with averages between 0.64 and $0.74\,\upmu \hbox {m}$.

### Monkey brain

From the *superior longitudinal fascicle*, we had only samples from the right hemisphere (Fig. 11a). The distribution was narrower than in the human brains, and the average diameter of $0.51\,\upmu \hbox {m}$ was smaller than in the corresponding hemisphere of the human brains ($p<0.001$).

This was not the case in the region of the uncinate/superior occipitofrontal fascicle (Fig. 11b). In the right hemisphere, the average was $0.55\,\upmu \hbox {m}$, i.e. quite similar to that in human brains. In the left hemisphere, the average was 0.78 $\upmu \hbox {m}$, significantly larger than in our human samples ($p<0.001$). Also, in contrast to the human brains, average diameter in the left hemisphere of this region was larger on the left than right side ($p<0.01$).

In the *corpus callosum*, distribution was also similar to that in human brains; the average in samples from the truncus, shown in Fig. 10c), was 0.58 $\upmu \hbox {m}$, close to that in the human brains.

### Thickness of myelin sheath

**Fig. 12:**
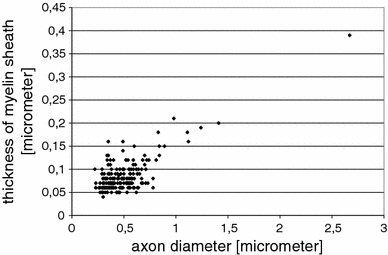
Thickness of myelin sheath plotted against inner diameter of axons, measured in the superior longitudinal fascicle of the monkey. The correlation coefficient is 0.75; the average thickness of the myelin sheath is 0.09 $\upmu \hbox {m}$, the median 0.08 $\upmu \hbox {m}$

In one of the monkey samples from the superior longitudinal fascicle, we measured the thickness of the myelin sheath in relation to the inner diameter of axons (Fig. 12). A clear correlation between thickness of myelin sheath and axon was found for those fibres with calibres above $0.6\,\upmu \hbox {m}$, not seen for fibres of smaller calibre. Overall average thickness of the myelin sheath was $0.09\,\upmu \hbox {m}$.

## Discussion

The brain contains vast numbers of interconnected neurons that constitute anatomical and functional networks. Typically, neural networks are characterized by nested self-organization, ill-defined operational units of strongly recursive nature, dependent on initial conditions, due to plasticity and evolution. The function and dysfunction of such, so-called complex dynamic systems, can only be understood by studying extensively their micro-, meso- and macro-connectivity. Not surprisingly, therefore an increasing number of investigators employ a variety of invasive (e.g. histology, ECoG) and non-invasive (e.g. EEG, MEG, TMS, MRI) methodologies in order to describe brain connectivity at different spatial and temporal scales.

One such methodology is that of investigating axonal calibres in different cortico-cortical or sub-cortico-cortical bundles. Indeed, the distribution of axonal diameters is an outstanding characteristic of network topology, hinting upon the scales of organization of widespread neural networks.

Here we used electron microscopy to investigate the distribution of calibre of cortico-cortical axons in the superior longitudinal fascicle, and the uncinate/inferior occipitofrontal fascicle at the transition between temporal and frontal lobe. These (and some other) fascicles run in the very depth of the white matter and carry fibres which run over long distances, as known from dissections of the white matter (e.g. Gluhbegovic and Williams 1980). Numerically, these deep fascicles contribute only about 2 % of the cortico-cortical fibres connecting one hemisphere with itself through the white matter (Schüz and Braitenberg 2002). The vast majority of cortico-cortical fibres runs in more superficial regions, such as the U-fibre system, and connects neighbouring regions to each other. We found that inner diameters of myelinated axons in these bundles range from $0.16\,\upmu \hbox {m}$ and up to $9\,\upmu \hbox {m}$, and with it likely conduction velocities. The size distribution was skewed with the majority of axonal calibres averaging below $1\,\upmu \hbox {m}$ diameter and a small population of much thicker fibres. Since all of these fibres may be assumed to run over long distances, i.e. connecting different lobes or distant regions within one lobe, it is improbable that this distribution reflects only the distribution of fibre lengths; it rather suggests that fast conduction times in the long-range system are only required for particular functions. The discussion that follows gives detail of the results we obtained, their interpretation and their contribution in previous investigations.

### Conduction velocities, myelination and further aspects of fibre calibre

It is well established that the range of calibres bears a direct relation to the range of conduction velocities. For diameters of 10 $\upmu \hbox {m}$, a velocity of about 50 m/s has been measured in the peripheral nervous system of the cat (Hursh 1939). For cortical axons in monkeys, conduction velocities up to 27 m/s have been reported (Swadlow 2000). Wang et al. 2008 came to the conclusion that maximum calibre of cortical axons increases so as to allow similar minimum conduction time over the cortex of 1–5 ms, independent of brain size. For the human brain, in which long fibres may reach lengths of up to 15 cm or so, conduction times of 5 ms would require a conduction velocity of 30 m/s. With the maximum fibre diameter of $9\,\upmu \hbox {m}$ found here, this rule may also apply to the human brain and explain some of the time preservation with brain size, mentioned in the introduction.

At the lower end, it is expected that, in addition to small myelinated fibres, a proportion of unmyelinated fibres may also exist in the human cortical white matter. In our material, unmyelinated fibres were not preserved, but their existence is well documented in the cortical white matter of various mammalian species (Partadiredja et al. 2003; Berbel and Innocenti 1988). In parts of the corpus callosum, the percentage of unmyelinated fibres was found to be around 30 % in such different species as rat, cat, horse and cow (Olivares et al. 2001), as well as macaque (Swadlow et al. 1980; LaMantia and Rakic 1990; Wang et al. 2008; for overviews see Innocenti 1986; Olivares et al. 2001). In contrast to this constant value over various species, there is, however, also good evidence for a correlation between percentage of myelinated fibres and brain size from a study by Wang et al. (2008). Concerning the human brain, Aboitiz et al. (1992) report 16 % unmyelinated fibres in the genu of the Corpus callosum, but usually less than 5 % in other regions of the Corpus callosum. The human brain which they had at their disposal was fixed relatively shortly post-mortem which still enabled them to distinguish between myelinated and unmyelinated fibres.

Unmyelinated axons have conduction velocities below 1 m/s, as documented for the peripheral nervous system (Hoffmeister et al. 1991). The lowest conduction velocities in ipsilateral cortico-cortical axons in the rabbit have been found to be 0.21–0.4 m/s (Swadlow 2000). The slowest reported antidromic conduction velocities for central axons (measured in the olfactory system of the rat) are about 0.1 m/s (Ferraya Moyano and Molina 1980). Similar conclusions have been reached using orthodromic conduction methods in slices of the rat motor cortex (Aroniadou and Keller 1993), of the monkey prefrontal cortex (Gonzaléz-Burgos et al. 2000), and in in vivo monkey experiments using visual stimulation (Bringuier et al. 1999).

Other important aspects of axon calibre and/or myelination have been pointed out in the seminal papers by Wang et al. (2008) and Perge et al. (2009, 2012), such as the relation to energy consumption, firing rate, information rate or size of terminal arbours.

Our finding of a correlation between axon calibre and thickness of myelin sheath is in accordance with what has been found for the spinal cord in various vertebrate species (Hildebrand and Hahn 1978; Leenen et al. 1985) and for the peripheral nervous system in mammals (Gasser and Grundfest 1939; Williams and Wendell-Smith 1971; for review see Bischoff and Thomas 1975). In a recent study, it has been shown that myelination is not necessarily an all-or-nothing principle, but that myelinated axons can differ with respect to the length of the premyelin axonal segment and that myelination can sometimes be interrupted for up to 55 $\upmu \hbox {m}$ (Tomassy et al. 2014).

### Comparison with other electron-microscopic studies on the human cortical white matter

There are only few electron-microscopic studies on the human brain to which our data can be compared. One of them is the study by Aboitiz et al. (1992) on the corpus callosum of one human brain. Diameters in their case ranged from 0.2 $\upmu \hbox {m}$ up to more than 10 $\upmu \hbox {m}$, with the majority of fibres in most regions being below 1 $\upmu \hbox {m}$, similar to our findings. The medians in their study ranged between 0.6 and 1 $\upmu \hbox {m}$ and showed significant local differences along the anterior–posterior extent of the corpus callosum, both in the electron and light microscope (Aboitiz et al. 1992, 2003). These differences were also described in the light microscope study by Caminiti et al. (2009). In the two human brains in which we investigated the corpus callosum, medians ranged between 0.5 and 0.6 $\upmu \hbox {m}$ and did not show a statistically significant difference between sampling regions. However, local variations over the extent of the corpus callosum were not the focus of our study, and samples were taken from only three different places, in contrast to the studies of the aforementioned investigators. Local variations may therefore have escaped our notice.

Our measurements on the superior longitudinal fascicle may be compared to a clinical electron-microscopic study by Zikopoulos and Barbas (2010). It deals—among other things—with axonal diameters in fibres running anterior-posteriorly in the depth of the white matter beneath the lateral prefrontal cortex. They found averages of inner diameters (in different persons) around 0.5 and $0.6\,\upmu \hbox {m}$, i.e. close to our values which ranged between 0.6 and $0.7\,\upmu \hbox {m}$ in most cases (see Table 1).

In a recent electron-microscopic paper by Liu and Schumann (2014), axon diameters in the deep white matter underneath the superior temporal cortex were investigated. The overall distribution was similar to ours, and mean values of diameters in different individuals ranged between 0.8 and $0.85\,\upmu \hbox {m}$.

### Comparison between different cortico-cortical systems

The average diameters in the superior longitudinal fascicle did not differ significantly from those in the corpus callosum. In contrast, the average diameters in the uncinate/inferior occipitofrontal fascicle were smaller than in both the superior longitudinal fascicle and the corpus callosum. This may reflect the fact that the uncinate part of this fascicle mainly connects regions between the temporal and frontal pole which is a relatively short distance compared to the distance covered by some fibre populations in the superior longitudinal fascicle. However, the calibre of cortico-cortical fibres does not necessarily correlate with fibre length, but may differ between fibre systems, as has been shown in light-microscopic studies by Caminiti et al. (2009) and Tomasi et al. (2012) for the corpus callosum.

The positive skew characterizing the distribution of fibre diameters in the corpus callosum and in the cortico-cortical fascicles is neither restricted to humans nor to these systems. Positive skews have also been described in the corpus callosum of rodents, cats and primates (Olivares et al. 2001; Wang et al. 2008; Tomasi et al. 2012), in other commissures of the monkey (LaMantia and Rakic 1990), in other regions of the cortical white matter of rats and monkeys (Partadiredja et al. 2003; Innocenti et al. 2013) and in the spinal cord of humans and rats (Häggqvist 1936; Leenen et al. 1985). The topic of types of distributions is dealt with in detail in the study by Perge et al. (2012) who found positive skews also in the fornix, the optic and olfactory nerve and in parallel fibres of rodents and even invertebrate fibre tracts, but they also found a few systems with negative skews. For a comparative overview over properties of fibre systems in vertebrate brains see also Nieuwenhuys et al. (1998).

### Quality of the material: comparison with the monkey brain

The long intrahemispheric association bundles investigated here also exist in the monkey brain (e.g. Cowley 1983; Ungerleider et al. 1989; Yeterian et al. 2012). In order to compare our human tissue to well-fixed material, we included a monkey brain in this study.

The main difference between human and monkey material was the high packing density in the monkey compared to the low packing density in humans, due to the different quality of fixation. Lower packing density can be assumed to be partially due to the disintegration of cellular material as indicated by the debris in the space between myelinated fibres. However, differences in extracellular space may have additional reasons: an increase in brain weight of about 20 % is observed in human brains during the first 20 hours of fixation by immersion in formalin (Blinkov and Glezer 1968). It is quite possible that this contributes to an increase in extracellular space compared to tissue fixed by cardiac perfusion in animals. In addition, there is evidence that there is in reality more extracellular space in the intact brain than found in conventional electron-microscopic material (Cragg 1979; Hrabetová and Nicholson 2007). Thus, the low density of tissue found here and in other electron microscopic studies on the human brain does not necessarily imply a great loss of fibre populations. This is supported by the fact that, on the whole, distributions and mean values in axonal diameter were similar to those in the monkey brain. Thus, our results from the human brains can be considered to be reliable in spite of the late fixation inherent in studies on human post-mortem material.

A few other things distinguished the monkey from the human brain: the average diameter in the region of the superior longitudinal fascicle was smaller in the monkey. This is not astonishing in view of the smaller size of the monkey brain. However, the average calibre in the uncinate/inferior occipitofrontal fascicle of the left hemisphere was larger than in the human brains. The most probable explanation for the larger value in the monkey is that we did not hit exactly homologous regions within this bundle. Another difference was an asymmetry between left and right in this bundle which we did not find in the human brains. However, it is possible that comparison between species and hemispheres is complicated by the fact that local differences may exist within bundles. Evidence for this comes from a diffusion tensor imaging study by Park et al. (2004). They found in the human brain a larger fractional anisotropy in the right hemisphere in the middle and inferior portion of the uncinate fascicle and vice versa in the superior part of this bundle.

### Comparison with other mammals; possible role of axon calibre range in large brains

**Fig. 13:**
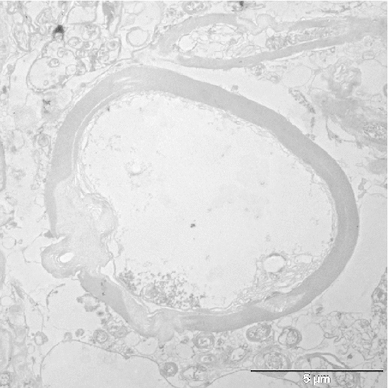
Largest axon found in our material (not contained in samples measured). It was located in the region of the superior longitudinal fascicle of human brain 2 (*left hemisphere*). *Bar* $5\,\upmu \hbox {m}$

It has also been shown in previous studies in mammals that the bulk of axons in the corpus callosum has similar calibres in large and small species. Large brains differ from small ones mainly by a lengthening of the tail of the distribution towards larger values, i.e. a small population of thick fibres is added in large brains (Schüz and Preißl 1996; Olivares et al. 2001; Wang et al. 2008). In our diagrams, a larger spread of fibres in the human than in the monkey brain is evident only in the superior longitudinal fascicle. However, when we did not restrict ourselves to random samples but searched actively for the largest fibres in our material, we also found them to be larger (up to $9\,\upmu \hbox {m}$; Fig. 13) in the human than in the monkey (up to $6.5\,\upmu \hbox {m}$).

The fact that the bulk of axons has a similar calibre in large brains as in small ones can be explained by the need to keep brain size within reasonable limits. An increase in the number of neurons leads to a more than proportionate increase in brain volume due to the necessary increase in fibre length (for review see Schüz and Sultan 2009). This would imply an increase in conduction times in large brains, unless compensated by additional increase in fibre diameter, as mentioned in the introduction. Ringo et al. (1994) showed the limitations of such a compensation: making conduction times as short in human brains as in small brains would lead to an impossibly large brain volume. Thus, it makes sense to increase the diameter of only those axons in which fast conduction velocity is really relevant, e.g. for movement detection or for maintaining dynamical properties in large brains (Buszáki et al. 2013).

Large brains obviously perform very well in spite of a majority of long-range axons having slow conduction velocities. Thus, as already touched upon in the introduction, an increase in conduction times with brain size may even be advantageous and related to the improvement of cognitive abilities. The role of delay lines for brain dynamics has been dealt with in various simulation studies (e.g. Pouille and Scanziani 2001; Roxin et al. 2005; Roberts and Robinson 2008; Bojak and Liley 2010). One important aspect is that the extent of temporal convergence within a neurone, for signals that start at different times in different presynaptic neurones, depends on the range of conduction times in the respective axons (“polychronization”, see Izhikevich 2006). To have a larger range of conduction times allows longer patterns in time to be recognized at a neuronal level. Convergence of different delay lines on single neurones has been suggested as a mechanism for distinguishing consonant speech sounds like b and p (Miller 1987), as well as for a variety of other perceptual and cognitive functions involving time intervals up to a few hundred milliseconds. These are functions involving exact timing, representation of exact temporal patterns or temporal integration over such intervals, and for which the left hemisphere often outperforms the right hemisphere (see for instance, Table 11 in Miller 1996). This led to a theory on lateralization based on the assumption of longer average conduction times in the left hemisphere than in the right (Miller 1996, 2008). It motivated us to compare left and right hemisphere, although it is clear that a difference in average conduction velocity may well be undetectable with our method in which the percentage of unmyelinated fibres could not be taken into account.

### Left–right differences

In one of the three human brains (brain no 1), we found indeed considerably larger axons in the superior longitudinal fascicle of the right than the left hemisphere as predicted by Miller’s theory which is in accordance with the finding by Büchel et al. (2004) of a higher anisotropy in the left arcuate fascicle. In the other two brains, we could find no significant difference between left and right with respect to axon diameters. Since we know nothing about language abilities or handedness in these brains or differences in the arrangement of sub-bundles between individuals, we cannot interpret the finding any further. However, individual differences between brains are always interesting. Individual differences with respect to asymmetry were also found for the *volume* of the superior longitudinal fascicle, as well as the uncinate fascicle (Bürgel 1999). It is to be expected that current improvements in diffusion weighted imaging (DWI) methods (e.g. Dyrby et al. 2013) will make it possible to deal with differences between hemispheres or between individuals in more detail and in larger populations in the future. Development of these methods so far is promising: the diameter of fibres in fixed material from the optic and sciatic nerve of the pig can already be demonstrated astonishingly well with DWI (Assaf et al. 2008). This method has also been used already to measure average axon diameters in the corpus callosum of the human brain in vivo. This gave estimated absolute values which were far too large compared to data from electron microscopy; however, relative differences in axonal calibre along the corpus callosum could be replicated (Alexander et al. 2010).

One more difference between left and right is noteworthy in this context: as evident from Table 1, in 5 of the 6 human left–right pairs, the number $n$ of axons measured is larger on the right than on the left. This prompted us to estimate the total area covered by the cross sections of the axons measured. It turned out that in case of all three human superior longitudinal fascicles and two of the uncinate fascicles, this area was larger in the right hemisphere. Although various components contribute to the area not included in our measurements, it is tempting to speculate that there was a larger proportion of “hidden” unmyelinated axons on the left which would be in line with the theory on lateralization mentioned above. Our methods prevent us from making a strong point on this. Further investigations with other methods are needed.
